# Rural Economy in Its Relations with Chemistry, Physics, and Meteorology

**Published:** 1845-07

**Authors:** 


I. RURAL ECONOMY IN ITS RELATIONS WITH CHEMISTRY, PHYSICS, AND METEOROLOGY. By *J. B. Boussingault*, Member of the Institute, &c. Translated by *G. Law*. 8vo. pp. 690. Baillière, London, 1845.

II. MANUAL OF AGRICULTURAL ANALYSIS. By *John Mitchell*. Small 8vo, pp. 143. London: Simpkin & Marshall, 1845.

THE subjects treated of in M. Boussingault's book are such as, at first sight, would appear hardly to entitle it to a place in our pages, but the rapid progress which the various departments of Organic Chemistry have recently made, and the important evidence which has been brought to light by the labours of chemists, give a great degree of interest to the progress of the science, and render their investigations into the chemistry of life, of the highest importance in a practical, as well as in a scientific, point of view. The author of the book now before us, is a chemist of high and deserved reputation, a laborious and careful experimenter, a cautious and accurate observer; and hence his views are well entitled to respect, even from those who differ from him in matters of science. The author's object is to place before his readers a complete system of scientific husbandry, in which the practical operations of the farmer are methodically arranged, and their principles minutely explained, by reference to the known laws of chemical and physical science. The majority of all farming operations have for their object the production of animal food, and consist more or less of processes of combination or transformation, in which organic matter is produced by the union of its elements. It is evident that, before these processes can be thoroughly understood, a great amount of preliminary information must be acquired. The chemical composition of muscular fibre, fat, and the various tissues and secretions of the animal body must be ascertained; as well as the composition of those substances which constitute the food of animals, and the changes which they are liable to undergo when placed in the stomach of an animal, before the true theory of animal nutrition can be established. Accordingly, for some time past, the inquiries of chemists have been directed to the study of those organic substances which constitute the food of animals, and very considerable progress has been made in explaining the hitherto little understood mysteries of digestion and assimilation.

At the same time, however, that chemists have thus been engaged in collecting facts, and recording valuable experiments, they have unfortunately in too many cases been led away from the simple search after truth, to follow up in all its ramifications some favourite theory or hypothesis, and have thus, as it were, lost the substance for the shadow, being more anxious to establish the correctness of their theory, than to ascertain the true cause of the phenomena it was originally intended to explain. But, notwithstanding this, physiologists have much to thank chemistry for, and every year, by increasing our knowledge of facts, carries us nearer to those great generalizations, which at once bring order and harmony, out of apparent confusion and disorder, giving us the means of explaining innumerable facts and phenomena by one simple and invariable law.

The last ten years have effected a most important change in the science of organic chemistry, not merely from the valuable discoveries which have been made in it, but likewise from the introduction of a more strict and logical system of inquiry, a nearer approximation to mathematical accuracy, than had hitherto been attempted, or even thought of. This has arisen from various causes, and amongst others, from the improved modes of analysis, first employed by Dr. Prout, and subsequently brought to perfection by Liebig, Dumas, Mitscherlich, Erdmann, Marchand, and Will. The result of this is, that the composition of an immense number of different vegetable products has been most accurately ascertained, and the modifications which they are liable to undergo, from the influence of temperature, oxidation, fermentation, or mere spontaneous decomposition, have been clearly made out: it is true that many of these changes are nearly as unintelligible as they were before, as far as the power which occasions them is concerned ; and various names have been given to that unknown power, which effects the transformation of substances, as it were, by the mere force of example, and in a manner quite different from that of all ordinary chemical affinity ; but, though the nature of the power itself is still involved in mystery, the effects which it produces are well understood, and the exact changes which it causes in the composition and properties of organic substances are well ascertained.

These enquiries have of course greatly facilitated the more complicated study of animal chemistry ; for, as a little consideration shews us, the food of all animals is derived directly or indirectly from the vegetable kingdom. The Herbivora feed on various proximate principles formed by plants, modifying or consuming some, and appropriating or assimilating others in an unaltered state. The Carnivora, in feeding on the flesh of other animals, take into their own systems the very substances, which the latter had assimilated or obtained from the vegetables which constituted their food. At the present time, though a considerable part of the chemistry of organic life is tolerably well understood, yet there is much of the theoretical part of the subject, and yet more of its practice, which is imperfectly, or not at all explained. Every discovery in any one branch of natural science shews more strongly the important bearing which the various sciences have upon each other, and makes it more evident that, in order successfully to study one, it is necessary to become acquainted with more than the rudiments of the others. Thus, in studying animal chemistry, we find it essential to take into consideration vegetable as well as animal physiology, the laws of heat, light, and electricity, the effects of climate, the composition of the atmosphere, and the laws of aeriform bodies ; the nature of those inorganic substances which form necessary components of the bodies of animals, or which exert remarkable influence upon them, as stimuli or poisons, and a multitude of other similar subjects. From the fact, that organic chemistry is even still, as it were, in a transition state, it is no easy task to write a good book upon the science; for, not only do many statements soon become unnecessary or erroneous, as new experiments bring fresh facts to light, so that what was a correct view of the state of the science at one time, becomes old-fashioned in a few months; but also, as many theories are still doubtful or disputed, it becomes necessary either to quote the various explanations which have from time to time been advanced, or else merely to state the facts, and leave others to draw their own conclusions. The latter course no doubt in many cases is the best, but it has this disadvantage, that it obliges the author to enter into a minuteness of detail, which renders his writings lengthy and tedious. M. Boussingault's book may, with reason, be called one of the best, if not the very best, of those which have recently appeared on the subject; it is written in a cautious philosophic spirit, and though in parts his style is verbose, yet this defect of manner may be readily pardoned, as throughout, the matter is good; in many places, especially where treating of those matters which have particularly been the subject of the author's own researches, there are passages full of value and interest.

In a book like "Rural Economy," which aspires to be a system of scientific husbandry, as will be supposed, there is little which has not already appeared elsewhere in print; but those researches which were hitherto only to be found in the various volumes of scientific periodicals, are here brought together, and arranged in a convenient and methodical form. The work commences with a chapter on vegetable physiology, the composition of plants, and their mode of growth. After describing a number of experiments on the germination of seeds and growth of young plants, the following conclusions are drawn.

"First.—That trefoil and peas grown in a soil absolutely without manure, acquired a very appreciable quantity of azote, in addition to a large quantity of carbon, oxygen, and hydrogen.

"Second.—That wheat and oats grown in the same circumstances, took carbon, hydrogen and oxygen from the air and water around them; but that analysis shewed an increase of azote in those plants after their maturity.

"The mode of experimenting followed had it in view simply to determine the assimilation of azote by certain vegetables, without entering into the question of the means by which this was effected, and indeed in reference to this point, I can only offer conjecture.

"Azote may enter the living frame of plants directly, or as M. Piobert has maintained, in the state of solution in the water, always aerated which is taken up by their roots. The observations of vegetable physiologists are not generally favourable to this view. It is farther possible that the element in question, may be derived from ammoniacal vapours which, according to some philosophers, exist in infinitely small proportion in our atmosphere. These vapours, dissolved by rains and dews, would readily make their way into plants, and might there undergo elaboration." P. 50.

In this passage the author seems, at first, inclined to throw doubt on the generally-received theory, of the ammoniacal origin of the chief part of the azote of plants; though, in so doing, he does not propose any new plausible theory, or advance any experiments to shew that this theory is founded in error. Indeed it is proved by what follows that this is not his opinion, he goes on to say :—

"It is long since Saussure alluded to the probable influence of ammoniacal vapours upon vegetation. Professor Liebig has more recently maintained the same opinion, and has taken particular pains to prove that rain water always contains a very minute quantity of carbonate of ammonia.

"To this cause, which must have the effect of infusing an azotised principle into the tissues of plants, must be added another, which is perhaps not the least energetic. It is this, that under certain electrical influences, of which M. Becquerel has made a particular study, hydrogen in the nascent state in contact with azote, may actually give rise to ammonia. By means of this view, it becomes easy to conceive how new azotised organic substances, under the mere influence of the putrid fermentation, might give origin to ammoniacal salts which would then exercise a fertilizing action on the soil." P. 50.

These remarks are interesting as bearing upon the formation of ammonia and other compounds of nitrogen in the soil, a subject of great importance in a theoretical, as well as in a practical point of view; but they have nothing to do with the assimilation of the azote of ammonia by growing plants. The facts are well known, that ammonia is formed during putrefaction, that it does exist in the air, and that it has a very remarkable effect on vegetation; and it is in consequence more than probable that much of the nitrogen of plants is derived from the decomposition of that substance. The second chapter contains an account of the proximate principles of plants, the observations on the azotised elements are very clear and excellent.

"Thus, according to M. Payen, the nutritious juices, which ascend from the extremities of the radicles to the terminal points of the leaves, carry an azotised principle which accumulates in all the growing organs, at the same time that it is deposited within the entire extent of the canals which the sap traverses. It might therefore be supposed that, in the latter situation, the azotised substance was associated with matters of ternary constitution, so as to form membranes and tissues. But from the various organs of the many species studied, M. Payen succeeded in dissolving out by means of alkalies, and entirely eliminating the animalized substances, without causing the slightest rent or erosion in the tissues perceptible with the microscope; whence it may fairly be concluded that if these substances everywhere and always accompanying the young tissues of plants, they still form no integral part of them. The animalized matter seems consequently to preserve a kind of independence with reference to the organs which secrete, which convey, and which contain it; it preserves a sort of mobility, which allows of its displacement, and it was in fact necessary that this should be so; for as the period of maturity approaches, we see the azotised substance carried more particularly forward towards the generative organs, and finally become fixed, as it were, and accumulated in the seeds. I have had frequent occasion to satisfy myself that trefoil, red beet, turnips, &c., contain much less azote after ripening their seeds than they did previously, and all husbandmen know that the straw or refuse of plants that have run to seed, forms very indifferent fodder for cattle." P. 90.

This is a happy combination of the deductions from scientific experiments, and the results of practical farming. In the third chapter the theory of fermentation, and that remarkable series of transformations which saccharine substances undergo in passing into spirit, are explained. The fourth chapter treats of soils, their structure, chemical composition, mechanical properties, formation, and classification; and contains a vast amount of useful information, which will be studied with advantage by all practical agriculturists. The composition of soils and the consideration of those alterations in their constitution which are produced by the growth of plants, naturally lead us to the subject of manures, and this is very fully treated in the fifth and sixth chapters; the former of which is devoted to organic, and the latter to inorganic manures. The following introductory observations will illustrate the extended view of the subject which the author takes, and the clear and simple manner in which he arranges the subject.

"All the agents employed by the agriculturist to restore, preserve, and augment the fecundity of the soil, I shall term manures. In my view, gypsum, marl, and ashes are manures, as much as horse-dung, blood, or urine; all contribute to the end proposed in employing them, which is the increase of vegetable production. The best manure, that which is in most general use, is precisely that which, by its complex nature, contains all the fertilizing principles required in ordinary tillage.

"Particular cultures may demand particular manures; but the standard manure, such as farm-dung, for example, when it is derived from good feeding supplied to animals with suitable and abundant litter, affords all the principles necessary to the development of plants; such manure contains at once all the usual elements which enter into the organization of plants, and all the mineral substances which are distributed throughout their tissues; in fact, carbon, azote, hydrogen, and oxygen are found therein united with the phosphates, sulphates, chlorides, &c.

"In order to be directly efficacious, every manure must present this mixed composition. Ashes, gypsum, or lime spread upon barren land, would not improve it in any sensible degree; azotised organic matter, absolutely devoid of saline or earthy substances, would probably produce no better effect; it is the admixture of these two classes of principles, of which the first is derived definitively from the atmosphere, whilst the second belongs to the solid part of the globe, which constitutes the normal manure, that is indispensable to the improvement of soils.

"Dead organic matter, subjected to the united influence of heat, of moisture, and of contact with air, undergoes radical modifications, and passes by a regular course of transformation into a condition more and more simple. The tissues, so long as they form a part of the animated being, are protected against the destructive action of the atmospheric agents; in plants and animals this protection is not extended beyond the period of their existence; destruction commences with death, if the accessory circumstances are sufficiently intense; and then ensue all the phenomena of decomposition, of that putrid fermentation which, at the expense of the primitive elements of the organised being, generates bodies more stable and less complicated in their constitution, and which present themselves in the gaseous and crystalline conditions, forms which are affected by the inorganic bodies of nature in general." P. 311.

M. Boussingault admits that plants derive a large proportion of the carbon which they contain from the free carbonic acid of the atmosphere, but he denies that this is the only source, observing that it is possible that certain elements of carburetted dungs may be directly assimilated. That there is some truth in this remark is more than probable, for there seems good evidence to prove that, in some cases at least, plants do absorb not only certain forms of hydrocarbon, but also small quantities of organic matters. The value of ammonia as an element of manure, is more proved, by reference to the results of practice, than by the support of scientific experiments; the author observes:—

"Agriculturists have, in all ages, admitted that the most powerful manures are derived from animal substances, an opinion or rather a fact, which, expressed in scientific language, amounts to this, that the most active manures are precisely those which contain the largest proportion of azotised principles. It is obvious, indeed, from everything which precedes, that all the substances which contribute to farm-dung contain azote; and that into many of them, such as uric acid, hippuric acid and urea, this element enters very largely.

"When we consider the immediate changes which all highly azotised substances undergo in the process of putrefaction, we can foresee that, in their transformation into manure, they must give origin to ammoniacal salts; and well-established facts prove beyond doubt that salts, having ammonia for their base, must be ranked among the most powerful of all the agents in promoting vegetation. It is sufficient, for instance, to bear in mind that, in the productive husbandry of Flanders, putrid urine is the manure that is employed with the greatest success; but we have seen that by putrefaction the urea of the urine is entirely changed into carbonate of ammonia. The fields of Flanders are consequently fertilized with a solution of carbonate of ammonia in water."

"Along a great extent of the coast of Peru, the soil, which consists of a quartz sand mixed with clay, and is perfectly barren of itself, is rendered fertile, is made to yield abundant crops by the application of guano, and this manure, which effects a change so prompt and so remarkable, consists almost exclusively of ammoniacal salts." P. 333.

It is, however, scarcely fair to adduce guano as a proof of the high value of ammoniacal salts, for though no doubt much of the effect produced by guano is due to the ammonia which it contains, we cannot suppose that all its value depends upon the presence of that substance. The fertilizing powers of guano depend rather on the joint effect produced by the phosphoric acid, ammonia, and azotised organic matters which it contains, and its value as a manure would be very greatly diminished were we to remove the phosphoric acid and azotised organic matters, which, by slowly decaying, present to the plants a constant supply of food. M. Boussingault's observations on the management of manure-heaps, and the use of liquid manure are excellent, and the chemico-agricultural descriptions of all the ordinary forms of organic manures, are plain, yet accurate and comprehensive. It is unnecessary to speak of the well-known experiments which he made in conjunction with M. Payer, the value of these in a scientific point of view is self-evident; and the means which they give of comparing the theoretical results of scientific experiments, with the practical results of the farmer, will every year become more appreciated. At the same time, however, whilst these experiments necessarily point out numerous trains of experimental investigation, it must be remembered that the views on which they are founded are still merely theoretical, and therefore that the practical man must not be disappointed if the results of his experiments are not always so favourable as theory had led him to expect.

In the chapter on mineral manures or "stimulants," as the word "amendements" is most erroneously translated, the English reader is struck by many passages which illustrate the marked differences existing between French and English farming. Some of the theoretical views advanced are open to question and doubt, but the analyses and positive facts described, are valuable and highly interesting. Speaking of marl, he says:—

"One sample of marl which we analysed, gave 0.002 of azote; another, from the Lower Rhine, gave rather more than 0.001 of the same element. It were, therefore, very proper in analysing marls, chalks, &c. to have an eye to their organic or azotic as well as to their mineral constituents; there can be very little question of the azotised elements being at the bottom of the really wonderful fertilizing influences of the marls of certain districts." P. 405.

That certain recent marls or calcareous muds, may contain enough nitrogen, materially to affect their value as manure is exceedingly probable, but we cannot help thinking that the powerful effects sometimes produced by particular marls, is due to very different causes; namely, the presence of phosphorus, the direct chemical influence of the lime itself, and the mechanical alteration which it causes in the soil. But little light is thrown by M. Boussingault's remarks on the action of the nitrates as manures; he quotes the experiments of practical men, to shew that these salts cause plants to grow with increased vigour, but that the market value of the crops so produced is diminished; and then proposes the theory of their action, as a problem to be solved by further experiments.

"This, however, is a point in physiology which may be put to the proof by experiment, and seems peculiarly worthy of being tested in this way. I have admitted it as extremely probable, that the azote of the azotised principles of plants, has its source either in the ammonia which is the special ultimate product of the organic manure we employ, or in the azote of the atmosphere, or in both simultaneously; but the opinion which should maintain that the ammonia derived from the organic constituents of the soil, passes into the state of nitric acid before penetrating the tissues of plants, would find support nearly in the same facts which I have quoted as favouring the former view. We have seen, moreover, in our general consideration on nitrification, with what facility the azote of ammonia undergoes acidification in certain circumstances, a fact from which an argument of much potency for the nitric acid theory naturally flows. I shall here add an observation to which I have up to this time, perhaps, attached too little importance. When M. Rivero and I examined the highly irritating and poisonous milky sap of the Hura crepitans, we had occasion to leave a considerable quantity of the water derived from the sap, after separating the caseum, to itself; by the spontaneous evaporation of this water we collected really a considerable quantity of nitrate of potash. Since this time, I have had occasion to note the same salt in the sap of several trees of the Tropics. In the fruit and leaves, however, I have never found more than very minute quantities." P. 420.

It is not a little remarkable, that this very fact, of the existence of nitrates in the juices of growing plants, is brought forward by Liebig as proof that nitric acid does not yield nitrogen to plants; this, however, is a mere assertion, and one moreover not altogether borne out by facts, the truth is, that the existence of nitric acid in plants supplies no proof at all, either in favour of, or against the theory of its decomposition. Under the head of Gypsum, and after describing Liebig's theory of its mode of action, M. Boussingault proceeds to examine critically the probable truth of Liebig's explanation.

"Our harvest of clover, taken as dry, amounts on an average, from land strongly manured with gypsum, to 2 tons 1 cwt. very nearly, per acre; and this quantity agrees pretty well with that which appears common in Germany. It is generally allowed that by using gypsum we double the produce. It would follow from this, that an acre which had not received gypsum would yield no more than 20½ cwts. of dry clover; in my opinion the reduction would be still greater. Dry clover hay, made from the plant cut when in flower, contains about 2 per cent. of azote. The 20½ cwts. of forage gained by the intervention of the gypsum would consequently contain 110 lbs. of ammonia equivalent to 134·2 lbs. of carbonate of ammonia. This consequently is the quantity of carbonate of ammonia which the gypsum ought to have been the means of procuring from the rain which falls upon an acre of land during the time that clover is upon the ground, in order to furnish the azote contained in the increased quantity of the crop.

"Now in Alsace, from the time of applying the gypsum in April, to the time of mowing in July, there falls on an average 3·92 nearly 4 inches of rain, which would amount in round numbers to 982 tons per acre. When the azote of what may be spoken of as the surplus produce derived from the rain, in fact all the water that falls ought to contain $\frac{1}{17000}$ of its weight of carbonate of ammonia. It is very questionable, however, whether any such proportion of ammoniacal salts exists in rain water; yet the proportion ought to be very much greater, in as much as we have supposed the whole of the rain that fell to penetrate the ground, none of it to run off; but the truth is that a very considerable proportion of the rain that falls never sinks into the ground; once the surface is thoroughly soaked, much that falls drains off, passes away by the ditches, and is lost with all it may contain that would prove beneficial to vegetation." P. 430.

This kind of argument, does not, however ingenious, carry much weight, and is hardly ever a fair representation of the true conditions of the phenomena it is intended to illustrate; in the present instance, the original theory was, that gypsum fixed the ammonia otherwise in a free and volatile state, contained in the atmosphere, brought down by rain, condensed with dew, or escaping from the decomposing azotised matters contained in the soil; and not merely, as our author states it, brought down in rain-water. The more practical arguments which he next brings forward, as to the comparatively trifling effects produced under certain circumstances by gypsum, have far more weight than the preceding calculations, and lead our author to the conclusion, that the chief value of gypsum as a manure is, in supplying lime to the growing plants; his arguments are ingenious and plausible, but not entirely convincing. Both when treating of the use of gypsum, and likewise subsequently, when speaking of the use of ammoniacal salts as manure, M. Boussingault expresses himself convinced, that it is impossible that ammoniacal salts in which the acid is inorganic, excepting the carbonic, can be useful to plants, and he attempts to illustrate this by some experiments of M. Schattenmann, which prove that the quantity of sulphur and chlorine in the ashes of certain plants was much less than it must have been had the ammonia been wholly absorbed as a sulphate or muriate. It is surely no argument to say, that as all the ammonia could not have been so absorbed, therefore it is evident that none of it entered the plants in that form; and setting this aside, the experiments quoted are by no means unexceptionable, as it is open to doubt whether a notable quantity of sulphur, chlorine, &c., may not have been dissipated in the preliminary process of reducing the plants to ashes?

The seventh chapter contains a multitude of curious experiments in the rotation of crops, and is peculiarly valuable as the results described in it, being the actual operations of a farm of considerable magnitude, are free from those local effects and interferences which so greatly diminish the value of all agricultural experiments performed on a small scale. The eighth chapter explains the chemistry of feeding and fattening cattle, and, like the preceding one, invaluable for the practical experiments which it contains; the author agrees with most chemists in the view which he takes of the general nature of food.

"These several considerations, therefore, induce me to conclude that the nutritious principles of plants and their products reside in their azotised principles, and consequently that their nutritious powers are in proportion to the quantity of azote they contain. From what precedes, however, it is obvious that I am far from regarding azotised principles alone as sufficient for the nutrition of animals; but it is a fact, that very highly azotised vegetable nutritive substance is generally accompanied by the other organic and inorganic substances which concur in nutrition. (P. 521.)

The peculiar views held by M. Boussingault and M. Dumas on the subject of fattening, as opposed to those entertained by M. Liebig, are well known.

"It appears at first sight most opposite to Nature to suppose that the feeding ox finds the whole of the fat he lays on, ready formed in the food he eats; it is only, in fact, after having made repeated analyses of plants, and discovered fatty matters almost everywhere, and in quantities generally superior to any that had been suspected in the composition of plants, that the idea begins to acquire likelihood; finally, the chemist becomes convinced that it is so when he finds a regular association of neutral azotised substances and fatty principles in all the articles usually employed as food for cattle—in the grasses and cereals, in the leaves, stems, and seeds of plants.

"Fatty substances appear to be principally formed in the leaves, where they frequently shew themselves under the form, and with the properties of wax. Taken into the bodies of animals, mingled with the blood, and exposed to the influence of the oxygen of the inspired air, they will undergo an incipient oxidation, whence will result the stearic or oleic acid that is found as a constituent of suet. By undergoing a second elaboration in the bodies of the carnivora, the same fatty substances, oxidated anew, would produce the inorganic acid which characterise their fat. These divers principles, by a still further degree of oxidation, would give rise to the fat volatile acids, which make their appearance in the blood and in the perspiration. Finally, did they suffer complete oxidation, *i. e.* combustion, they would be changed into carbonic acid and water, and be in this shape eliminated from the economy." P. 564.

At the same time, however, he does not deny the possibility of fat being *formed* in the animal system; for he says—

"For my own part, I adopt the view which supposes an animal to be supplied with fat already formed, mainly because it presents itself to me as more in harmony with the facts which I observe in our stables. Still I do not deny that it may be possible for a certain quantity of fat to be elaborated in the bodies of herbivorous animals under the influence of a special fermentation of the sugar which forms an element in their food; although I feel satisfied, from practical facts, that sugar plays no essential part in the fattening of cattle." P. 566.

"All these facts are in such perfect harmony with the simple view of assumption and assimilation of fatty matters, that it is difficult to conceive on what foundation the opinion can repose, which would have them composed out of their elements in the animal body. Nevertheless, I am myself the first to admit that more extensive experience may lead to the modification, or even entire change of the opinion which I advocate. The facts on which that opinion is based, despite their number, are not probably yet sufficient to constitute a perfectly satisfactory or conclusive theory." 578.

We must confess, on the whole, that the arguments adduced in favour of the absorption theory of fattening are by no means strong, and it would almost appear that M. Boussingault himself feels this, by the cautious and reserved tone of some of the above observations.

Nothing can be more beautiful and complete than the series of experiments which prove the identity of composition of the azotised principles of plants and certain forms of animal matter; and this great point being once proved, it will at once be admitted, that very little change can be required to fit those substances for immediate absorption into the animal system. It is found that plants contain a considerable quantity of a substance or substances identical in nature with animal matter: these substances are absorbed direct into the system; independent of all evidence, it is surely improbable that these azotised substances in vegetable food should be transformed or consumed by the animal. Flesh has to be formed, the food contains the elements of flesh nearly formed, can we doubt then, that these elements are at once absorbed? in place of being consumed by respiration, whilst other vegetable matters are being transformed into azotised compounds, in fact, into the elements of flesh. Every known fact in animal chemistry is opposed to such a supposition. When, however, we search for evidence of a similar arrangement with regard to fat, we find probability strongly against such a theory. It is true that many plants contain large quantities of fatty or oily matters, very nearly allied, nay in some cases identical, with the fat of animals; but, on the other hand, we find many varieties of animal food, and amongst them some of those best fitted to fatten animals, almost wholly devoid of oil or fat of any kind; when digested in Ether, they yield a minute quantity of resin or wax, but no true fat. In these cases it is evident that the fat formed must either be derived from the transformation of some of the non-azotised principles of the food, such as starch, &c., or, supposing it present in sufficient quantity, from the wax or resin; in the latter case, as in the former, a distinct chemical transformation must take place; and if the necessity of this is once admitted, it does away to a great extent with the apparent probability of the theory, which supposes the fat to be *formed* by plants and *appropriated* by animals, whereas, if any chemical transformation is necessary, it is just as likely that starch or sugar may be changed into fat, as that resin may undergo such a transformation.

Although we do not think that M. Boussingault makes out a strong case in favour of his theory, it must nevertheless be admitted, even by those who adopt the "formation" view of the subject, that the oil or fat of vegetables may, in some cases, be absorbed by animals. The truth, as far as the evidence before us would shew, appears to be, that animals fed on oily food can appropriate and convert such fat to their own use, but that, quite independent of this, they possess the power of transforming non-azotised vegetable matters into fat.

The last two chapters of M. Boussingault's book treat of the more mechanical and statistical details of feeding and rearing stock; and the influence of meteorological phenomena on farming operations, a most important practical subject, and one which has hitherto been very much neglected by scientific agriculturists. Altogether the book is one highly deserving of praise, full of useful facts and sound information, and freer from wild theoretical speculation than the writings of most agricultural chemists.

In conclusion, it is much to be regretted that more care and attention has not been bestowed on the translation, it seems to have been hastily and imperfectly done, and in consequence contains numerous inaccuracies.

One of the many results of scientific farming, is the necessity which has arisen for a number of agricultural chemists. New chemical manures became the fashion, and accordingly chemists were required to assist in their manufacture. In a short time, however, dishonest persons found it more profitable to mix sand and all sorts of impurities with the chemical manures which they sold, than to send them out pure, and then a second set of chemists were required to detect and expose the frauds thus practised on simple and unsuspecting farmers. When it was found that a ship-load of brick-dust watered with stale urine, and called guano, was eagerly purchased at ten per cent. below the usual market price of that article, it is not surprising that some people found little or no effect from the use of guano, whilst others found even a small quantity killed their crops. It then became the custom to sell guano and other chemical manures with a certified analysis from an agricultural chemist; and this, for a time at least, put a stop to the system of plunder. It would appear, however, that even this arrangement was not free from defect, because, as manure-doctors had been found dishonest enough to prepare adulterated manures, so there were analysers, who did not scruple to assert that pure, which they knew was adulterated. Guano was sold with certificates stating that it contained 20, 30, or 40 per cent. of ammonia; and, at last, it was even offered with 70 per cent. of that substance! Now ammonia, being in its simple state a gas, always exists in guano in combination with some acid, let us for the time suppose it combined with carbonic acid: according to Phillips' analysis, common sesqui-carbonate of ammonia contains 29 per cent. of ammonia, so that, if the ammonia of this guano was present as a carbonate, it follows that the guano contained, independent of all other substances, at least two hundred and forty per cent. of carbonate of ammonia.

The absurdity of such a statement is self-evident to a chemist, but how is the farmer, who is wholly ignorant of chemistry, to perceive the utter uselessness of such certificates? The answer given by many to this question is, we must teach the farmer enough chemistry that he may be able to test the purity of his manures himself. This may do very well for experimental gentlemen farmers, who, trusting all the details of their farms to their bailiffs, have plenty of leisure to read scientific books, and make chemical experiments, but it is wholly out of the power of working farmers, who in fact can do nothing more than trust to the advice of any respectable chemist whom they happen to have an opportunity of consulting. During the last few years a number of books have been written on the subject of agricultural chemistry, all more or less intended to convey useful information to practical men, to enable them to understand and apply the laws of chemical science, to teach them chemical analysis, and to instruct them in the true mode of performing experiments in a reasonable and philosophical manner. In those books, the author's fear generally has been, that he should say too much; and that subjects of consequence might be passed over, or imperfectly treated, to make room for other matters of less importance; his aim of course has been to clothe his matter in the fewest possible words, and render his language as terse and concise as was compatible with simplicity and perfect comprehensibility.

Mr. Mitchell's Manual of Agricultural Analysis is a plain and well written little book on chemical manipulation and analysis, but it is not one which will be of much use to practical farmers, it is more suited for scientific country gentlemen, or village apothecaries, who have leisure to turn their attention to the subject of agricultural chemistry. Farmers who are accustomed to operate wholly on tons and cart-loads, are as unfit for delicate chemical experiments on fractions of grains, as they are unable to perform them from want of time. Mr. Mitchell's manual contains drawings of all the simple apparatus requisite for ordinary chemical experiments, together with plain and simple directions how to use them, the mode of using and applying tests, the nature of those elementary substances which are of most importance to the agricultural chemist, the origin and composition of soils, and the rudiments of analysis. The following brief extract, which illustrates the author's style, is from the first Chapter, on the Utility of Chemical Analysis.

"Farmers knew that certain manures were good for certain soils; they also knew that certain soils produced luxuriant crops of some vegetables, whilst others would not grow upon them; and they also knew that, on certain pastures, cattle thrived better than on others; *but they did not know the why and the wherefore*, and could not produce certain crops at will, nor did their manures always produce the same effect, although laid apparently on the same kind of land (weather, of course, being favourable). In this dilemma, the chemist steps in, and by informing the farmer the composition of all his crops, and then, by analysing the soil on which it is intended to grow any particular one, it can be readily ascertained whether it is fitted for the purpose. If it contains all the ingredients the crop requires, it will do; if not, they must be added in the shape of manure. And here, again, the chemist is required; for, if the manure be added without a knowledge of its contents, it may do more harm than good, by giving to the soil principles which are already in excess, and which are then deleterious to vegetable life.

"During the time other arts dependent on chemistry have been improved almost to perfection, agriculture, which is, without a single exception, the most ancient, the most universal employment of man, is still to a great extent, an empirical art. The experience of centuries has pointed out the benefits which result from the application of certain manures or 'fertilizers' to the land; but the farmer has, until very lately, taken no pains to inquire into the mode in which these various substances act. In some cases he applied gypsum to his land until it ceased to prove a benefit, and then he imagined he fully accounted for this circumstance by saying that the land was *tired* of gypsum. In other cases, salt has been applied with like result, and with the same pseudo-explanation." P. 3.

Every one who is at all acquainted with the science of chemistry must feel convinced that it will in time present a key to all the difficulties of farming, and solve all the problems of rural economy; but, at the same time, whoever studies the present state of agricultural chemistry, must perceive that chemists are by no means prepared to answer all the questions which farmers may wish to ask them; the composition of soils, crops, and manures is not yet fully investigated; the *why and the wherefore* is in truth not yet completely understood, and it will be still some time before it is: and that this must be the case no one can be surprised at who considers of how recent a date the whole science of agricultural chemistry is, and how important and extensive a subject it is. One other point in the above little extract demands comment, namely, the assertion that a soil containing the requisite elements must be fertile and fit for the growth of plants; this is not quite true, it is not only necessary that these substances be present in the soil, but likewise that they exist in that form and condition best fitted for absorption by plants; the mechanical structure and properties of the soil are quite as important as its chemical composition, and the very same elements, arranged in different conditions, may constitute a fertile or a comparatively barren soil.

